# Case Report: Eosinophilic granulomatosis with polyangiitis presenting as a multiple sclerosis mimic

**DOI:** 10.3389/fimmu.2026.1926608

**Published:** 2026-10-19

**Authors:** Benjamin Landau, Michael Kettner, Mathias Fousse, Myriam Metzger, Yoo-Jin Kim, Sergiu Groppa, Wenlin Hao

**Affiliations:** 1 Department of Neurology, Saarland University, Homburg, Germany; 2 Department of Neuroradiology, Saarland University, Homburg, Germany; 3 Institute for Pathology, Saarland University Medical Hospital, Homburg, Germany

**Keywords:** asthma, Churg-Strauss Syndrome, demyelinating diseases, eosinophilia, multiple sclerosis, vasculitis

## Abstract

Eosinophilic granulomatosis with polyangiitis (EGPA) rarely involves the central nervous system and may closely resemble inflammatory demyelinating disease. We report a 32-year-old woman with adult-onset asthma and recurrent sinusitis who presented with bilateral lower-extremity sensory symptoms and neuropathic pain. Brain and spinal magnetic resonance imaging (MRI) demonstrated multifocal T2-hyperintense lesions fulfilling dissemination in space and time according to the 2017 McDonald criteria. Cerebrospinal fluid (CSF) analysis revealed mild pleocytosis, CSF–restricted oligoclonal bands, and eosinophils on qualitative cytological examination. Multiple sclerosis (MS) was initially suspected and intravenous methylprednisolone resulted in partial improvement. Three weeks later, the patient developed palpable purpura, inflammatory arthralgia, and left foot-elevating paresis. Follow-up MRI demonstrated a new enhancing lesion and electrophysiological studies revealed axonal neuropathy. Retrospective review of the initial laboratory results showed that peripheral eosinophilia was already present at baseline, while subsequent evaluation identified eosinophilic gastritis on gastroscopic biopsy and eosinophilic bone marrow infiltration. The diagnosis of EGPA was established based on the clinical presentation, laboratory findings, and histopathological evidence of eosinophilic inflammation, and the patient fulfilled the 2022 ACR/EULAR classification criteria for EGPA. Treatment with corticosteroids and cyclophosphamide followed by maintenance mepolizumab resulted in sustained clinical stability. This case highlights that EGPA may present with radiological and CSF–findings suggestive of MS and emphasizes the importance of integrating systemic manifestations to avoid diagnostic delay.

## Introduction

Eosinophilic granulomatosis with polyangiitis (EGPA), formerly known as Churg–Strauss syndrome, is a rare systemic vasculitis classified within the spectrum of antineutrophil cytoplasmic antibody (ANCA)-associated vasculitides (AAV) (1). It is characterized by eosinophil-rich inflammation and small- to medium-sized vessel involvement, typically occurring in the context of asthma, eosinophilia, and systemic organ manifestations (2–4). However, unlike granulomatosis with polyangiitis and microscopic polyangiitis, ANCA are detected in only a subset of patients with EGPA, with MPO-ANCA positivity reported in approximately 30–40% of cases (1, 5). ANCA-positive and ANCA-negative EGPA may also show partially distinct clinical phenotypes, although substantial overlap exists. Importantly, the absence of ANCA does not exclude the diagnosis of EGPA, and ANCA status should therefore be interpreted in conjunction with the clinical, laboratory, radiological, and histopathological findings (5).

Neurological involvement occurs in approximately 50–60% of patients and most commonly affects the peripheral nervous system, particularly in the form of mononeuritis multiplex or other forms of peripheral neuropathy (3). Central nervous system (CNS) involvement is less common, but its reported frequency varies substantially among cohorts. In the French Vasculitis Study Group cohort, CNS involvement was reported in approximately 5% of patients, whereas a Chinese retrospective cohort of 110 patients reported a frequency of 17.3% (6, 7). In the latter cohort, cerebrovascular manifestations, particularly ischemic lesions, were the predominant CNS manifestations (7). Other reported CNS manifestations include hemorrhagic lesions, encephalopathy, and, more rarely, inflammatory or demyelinating-like lesions (6–8).

Multiple sclerosis (MS) is a chronic inflammatory demyelinating disease of the CNS characterized by focal inflammatory lesions disseminated in space and time (9). Clinically, MS typically manifests with episodic neurological deficits involving different CNS regions, including sensory, visual, motor, cerebellar, and brainstem dysfunction, often followed by partial or complete recovery. Typical magnetic resonance imaging (MRI) findings include T2-hyperintense lesions in periventricular, juxtacortical/cortical, infratentorial, and spinal cord regions, with gadolinium enhancement indicating active inflammatory lesions (10). Cerebrospinal fluid (CSF)-restricted oligoclonal bands provide evidence of intrathecal immunoglobulin synthesis and represent an important supportive biomarker in the diagnosis of MS (11, 12). Although these radiological and CSF findings are characteristic of MS, they are not entirely disease-specific and may occasionally occur in other inflammatory disorders of the CNS (11, 12).

The pathophysiological mechanisms underlying CNS manifestations of EGPA differ from those of MS but may result in overlapping clinical and radiological phenotypes. In EGPA, neurological injury may arise from eosinophil-rich inflammation, small-vessel vasculitic injury with secondary ischemia, and potentially inflammatory or demyelinating mechanisms (2, 3). In contrast, MS is primarily characterized by immune-mediated inflammatory demyelination within the CNS (13). The mechanisms responsible for the rare demyelinating-like CNS lesions reported in EGPA remain incompletely understood (3, 8).

Because inflammatory or demyelinating-like CNS lesions are uncommon in EGPA, their occurrence may create a diagnostic challenge when MRI findings fulfill dissemination in space and time and CSF-restricted oligoclonal bands are present (8, 14). Infrequently, EGPA may therefore closely mimic MS, particularly when systemic manifestations are subtle or initially considered unrelated to the neurological presentation (8, 12). Recognition of eosinophilia, asthma, sinonasal disease, skin manifestations, peripheral neuropathy, or other systemic inflammatory features may provide important clues to an underlying systemic eosinophilic disorder (2, 4).

We present a case of EGPA initially interpreted as MS because of demyelinating-like lesions on MRI and CSF-restricted oligoclonal bands. The subsequent emergence and recognition of systemic eosinophilic and vasculitic manifestations prompted diagnostic reassessment. This case highlights the importance of integrating CNS findings with the broader systemic phenotype when evaluating patients with an apparent inflammatory demyelinating disorder.

## Case description

A 32-year-old Chinese woman presented in May 2022 with a 10-day history of sensory disturbance in both lower limbs and burning pain in the right leg and both feet. She also reported postprandial abdominal discomfort for three days. Neurological examination revealed hypesthesia in the right leg and both feet. Muscle strength and muscle stretch reflexes were normal and symmetrical, and there was no evidence of a sensory level suggestive of transverse myelopathy. Bladder and bowel function were normal. Her medical history included asthma, first diagnosed at age 20, and recurrent sinusitis.

Initial brain and spinal MRI demonstrated multiple small T2-hyperintense lesions involving the periventricular and paraventricular white matter, juxtacortical regions (**Figure 1A**), left pons, bilateral midbrain, and spinal cord at the C3/4 and T7 levels (**Figure 1C**). Gadolinium enhancement was observed in a right temporal periventricular lesion, a right frontal juxtacortical lesion (**Figure 1B**), and the C3/4 spinal cord lesion. The T7 lesion was T2-hyperintense without reported contrast enhancement. The lesion distribution and enhancement pattern fulfilled MRI criteria for dissemination in space and time according to the 2017 McDonald criteria. Pansinusitis was also noted. Nerve conduction studies, tibial somatosensory evoked potentials, and visual evoked potentials were normal.

**Figure 1:**
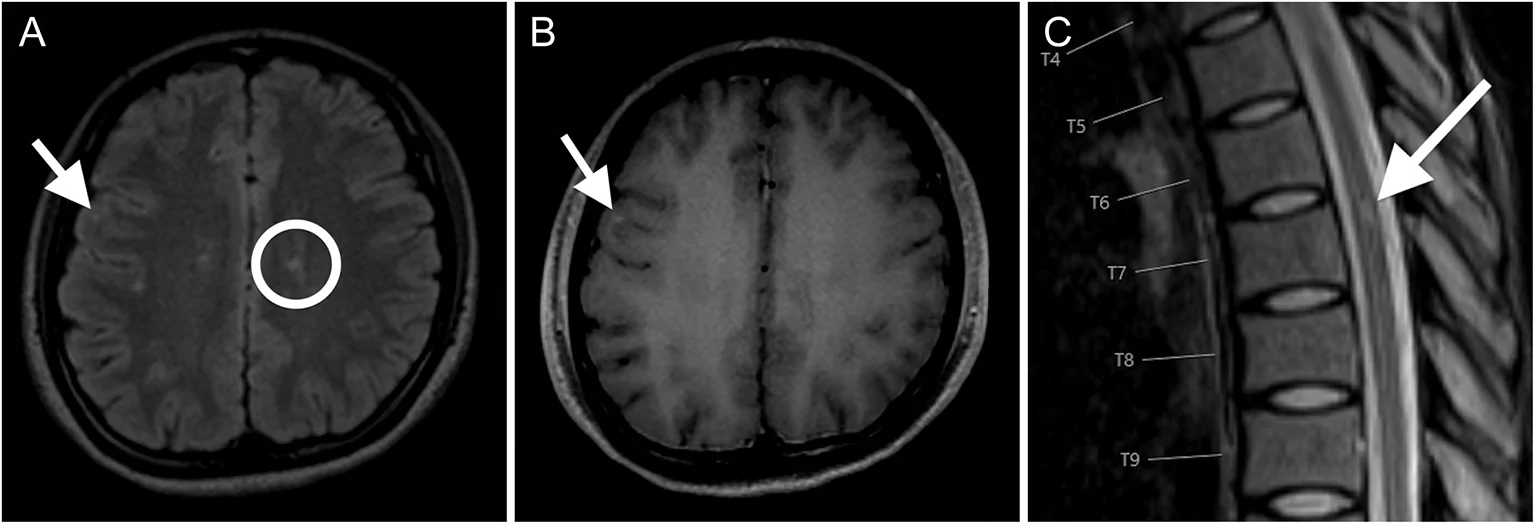
MRI findings demonstrating demyelination-like lesions in the patient with EGPA. Brain MRI was performed on a 1.5-T scanner. The native examination included T2-SPACE with multiplanar reconstructions, axial T2-FLASH, diffusion-weighted imaging with axial ADC mapping, and axial and sagittal T2-weighted sequences. Following intravenous administration of gadolinium contrast agent (Clariscan^®^), T1-SPACE with multiplanar reconstructions was acquired. Spinal MRI included native sagittal T2-weighted, T1-weighted, and T2-TIRM sequences, with additional axial T2-weighted imaging from T6 to T9. Following intravenous administration of Clariscan^®^, sagittal T1-weighted imaging was performed. **(A)** Axial T2-FLAIR image showing multiple hyperintense lesions in the left periventricular white matter (circle) and right frontal juxtacortical region (arrow). **(B)** Axial post-gadolinium T1-SPACE image demonstrating contrast enhancement of the right frontal juxtacortical lesion (arrow). **(C)** Sagittal T2-TIRM image showing a hyperintense spinal cord lesion at the T7 level (arrow), without reported contrast enhancement. EGPA, eosinophilic granulomatosis with polyangiitis; MRI, magnetic resonance imaging; ADC, apparent diffusion coefficient; FLAIR, fluid-attenuated inversion recovery; SPACE, sampling perfection with application optimized contrast using different flip angle evolution; TIRM, turbo inversion recovery magnitude.

CSF analysis revealed mild pleocytosis (8 cells/µL, reference range 0–5 cells/µL). Qualitatively cytological examination demonstrated eosinophils, erythrocytes and activated lymphocytes in the CSF. With the presence of erythrocytes, a contamination with peripheral blood cannot be completely excluded. Oligoclonal bands were present in CSF but absent in serum, consistent with Type 2 pattern. Regarding intrathecal immunoglobulin synthesis, CSF IgG was 2.49 mg/dL (reference range 1.00–3.00 mg/dL), while serum IgG was 1.80 g/dL (reference range 0.70–1.60 g/dL). The IgG index was 0.56 (reference value <0.65), which was within the normal range. Pathogen testing for neurotropic viruses, EBV, CMV, and JC virus was negative. Serum testing for aquaporin-4 and anti- myelin oligodendrocyte glycoprotein antibodies was negative. Complement levels were within the reference ranges, with C3c of 101 mg/dL (reference range 90–180 mg/dL) and C4 of 26.8 mg/dL (reference range 10–40 mg/dL). Anti-proteinase 3 (PR3) and anti-myeloperoxidase (MPO) antibodies were negative, with PR3-ANCA of 0.2 IU/mL (reference value <2.0 IU/mL) and MPO-ANCA of 0.6 IU/mL (reference value <3.5 IU/mL), respectively; cytoplasmic and perinuclear ANCA (c-ANCA and p-ANCA) were also negative. Rheumatoid factor (RF) was mildly elevated at 26 IU/mL (reference value <14 IU/mL), while ANA and dsDNA were negative. Soluble IL-2 receptor was mildly elevated at 768 U/mL (reference range 158–623 U/ml); ACE was normal (39 U/L, reference range 20–70 U/L).

Under the working diagnosis of relapsing–remitting MS, the patient received intravenous methylprednisolone (1 g/day for five days). Hypesthesia improved partially, but burning pain persisted. Disease-modifying treatment with fingolimod was recommended based on the clinical and radiological diagnosis of MS; however, fingolimod was not initiated.

Three weeks later, she re-presented with a palpable purpura on both lower extremities (**Figure 2A**), spreading from the feet to the calves, and painful swelling of the joints in both feet and the left hand. A new left foot-elevating paresis (Medical Research Council grade, MRC 4/5) was noted. MRI showed a new right parietal contrast-enhancing lesion (the corresponding images were not available for inclusion in this report). Motor nerve conduction studies showed reduced amplitudes of the left peroneal and tibial nerves with preserved conduction velocities, while the sensory response of the left peroneal nerve was not detectable. These findings were consistent with an axonal peripheral neuropathy. Laboratory testing demonstrated markedly elevated levels of RF (201 IU/mL) and soluble IL-2 receptor (1867 U/mL). Skin biopsy of the palpable purpura showed a largely unremarkable epidermal layer with minimal interface inflammation and a pronounced interstitial neutrophilic infiltrate extending into the deep dermis, with a partly perivascular distribution. No definite vasculitis was identified.

**Figure 2:**
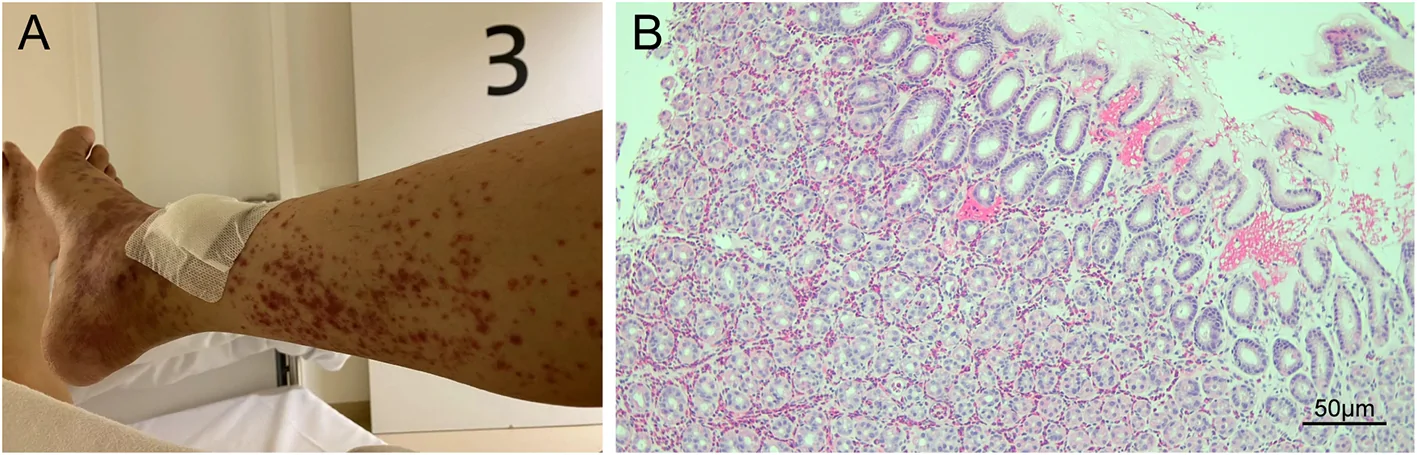
Clinical and histopathological features supporting EGPA diagnosis. **(A)** Clinical photograph showing palpable purpura on the lower leg during an acute vasculitic flare. **(B)** Gastroscopic biopsy demonstrating dense interstitial eosinophilic infiltration (H&E staining). Numerous eosinophils with characteristic pink cytoplasm are visible, in conjunction with clinical findings consistent with eosinophilic gastritis. EGPA, eosinophilic granulomatosis with polyangiitis; H&E, hematoxylin and eosin.

Given the clinical suspicion of vasculitis, a second course of intravenous methylprednisolone (100 mg/day for five days) was administered, followed by oral prednisolone tapering, resulting in complete resolution of the purpura, although a residual left foot-elevating paresis (MRC 4/5) persisted. After the initial corticosteroid therapy, the patient was advised to begin immunosuppressive induction treatment with cyclophosphamide. However, at that time, she was unable to decide whether she would proceed with cyclophosphamide. She was therefore continued on a tapering regimen of oral corticosteroids.

At the end of January 2023, after the corticosteroid dose had been reduced to 10 mg prednisolone per day, the patient reported increasing fatigue and severe upper-abdominal pain. Gastroscopic biopsy of the gastric antral mucosa showed segmentally marked interstitial eosinophilic infiltration of the stroma, without vascular association or definite extension into the epithelium. No *Helicobacter pylori* was detected on modified Giemsa staining, and no submucosa was included in the specimen. The findings were consistent with interstitial eosinophilia and, in the clinical context, supported a diagnosis of eosinophilic gastritis (**Figure 2B**); no histological evidence of vasculitis was identified. Bone-marrow biopsy confirmed marked eosinophilia (27.4%). Pulmonary function testing revealed an FEV_1_ of 74% predicted and an FEV_1_/FVC ratio of 72.5%; the ACQ-6 was not performed. Retrospective review of her initial laboratory results from May 2022 showed that eosinophilia of 33% (2.87 × 10^9^/L; reference ranges 0–5% and 0.02–0.50 × 10^9^/L, respectively) had already been present at first presentation. At that time, the patient was not taking any regular medication, making a drug-induced cause of the eosinophilia unlikely. Three weeks later, coinciding with the development of purpura, the eosinophil count had increased to 5.79 × 10^9^/L. Following corticosteroid treatment, the eosinophil count decreased to 2.12 × 10^9^/L.

An extensive diagnostic workup was performed to investigate secondary, infectious, malignant, and hematological causes of hypereosinophilia. Serological testing for parasitic infections, including *Schistosoma mansoni* and *Echinococcus spp.*, was negative. Computertomography (CT) of the thorax and abdomen showed no evidence of malignancy, and gastroscopy and colonoscopy revealed no evidence of gastrointestinal malignancy. Bone-marrow smear and biopsy showed marked eosinophilia but no evidence of leukemia; according to the available medical records, leukemia fusion gene screening and molecular testing related to acute myeloid leukemia (AML), myeloproliferative neoplasms (MPN), and myelodysplastic syndromes (MDS) were reportedly negative. Lymphoma immunophenotyping was also reportedly negative. Limited T-cell immunophenotyping showed elevated CD4-positive lymphocytes (2,050.98/µL; reference range 424–1,576/µL) and CD8-positive lymphocytes (957.12/µL; reference range 209–778/µL), with a CD4/CD8 ratio of 2.14. However, molecular testing for FIP1L1::PDGFRA and other myeloid abnormalities, as well as T-cell receptor clonality studies, was not performed. Serum IgE was not measured.

Sinus CT demonstrated chronic sinusitis. Cardiac evaluation showed a preserved left ventricular ejection fraction of 65% on echocardiography, and coronary CT angiography revealed no evidence of coronary involvement. Urine sediment showed no evidence of significant renal involvement, with 1 leukocyte/µL (reference value <25/µL), 2 erythrocytes/µL (reference value <23/µL), 0 cylinders/µL (reference value <1/µL), <5 bacteria/µL (reference value <1,200/µL), and 1 squamous epithelial cell/µL (reference value <31/µL).

According to the 2022 American College of Rheumatology/European Alliance of Associations for Rheumatology (ACR/EULAR) classification criteria for EGPA, the patient fulfilled the criteria for classification with a total score of 11 points: obstructive airway disease (+3), blood eosinophil count ≥1 × 10^9^/L (+5), mononeuritis multiplex/motor neuropathy not due to radiculopathy (+1), and extravascular eosinophilic-predominant inflammation on biopsy (+2) (4). No points were assigned for nasal polyps or hematuria, and the patient was negative for c-ANCA and anti-PR3 antibodies. The diagnosis of EGPA was established based on the overall clinical, laboratory, and histopathological findings, with the patient meeting the 2022 ACR/EULAR classification criteria for EGPA.

Given the severity and relapsing course of the disease and the presence of gastrointestinal involvement, a prolonged course of intravenous methylprednisolone was administered at a dose of 40 mg/day for six weeks, followed by an oral prednisolone taper. Cyclophosphamide was chosen as a guideline-concordant induction therapy for severe EGPA; rituximab represents an alternative induction option, but there is no established evidence that either agent is superior in this setting. Cyclophosphamide was initiated in February 2023 at 600 mg intravenously once weekly for four weeks. From March to August 2023, oral cyclophosphamide was continued at 100 mg every other day, followed by 50 mg every other day from September to October 2023, after which cyclophosphamide was discontinued. The cumulative cyclophosphamide dose was approximately 13.1 g. Mepolizumab was initiated in March 2023 at 300 mg subcutaneously once every four weeks and was continued as maintenance therapy until preparation of this report. Oral prednisolone was subsequently tapered to 2 mg/day and continued at this dose as maintenance therapy until manuscript preparation in October 2025.

During subsequent follow-up, no further clinical relapse or new systemic manifestations were observed, while residual left foot-elevating paresis persisted at MRC grade 4/5. Long-term MRI follow-up was not available because the patient declined further MRI examinations for financial reasons. Therefore, long-term treatment efficacy was assessed clinically rather than radiologically. **Table 1** summarizes clinical course, diagnostic assessment, treatment, and outcome.

**Table 1:** Timeline of clinical course, diagnostic assessment, treatment, and outcome.

Timepoints (month/year)	Clinical events/examinations/interventions	Findings/outcome
05/2022 Initial presentation	Bilateral lower-extremity sensory disturbance and neuropathic pain in patient with adult-onset asthma and recurrent sinusitis	Neurological examination: hypesthesia without motor deficit; normal reflexes; no sensory level
05/2022 Initial diagnostic work-up	Brain and spinal MRI	Multiple T2-hyperintense periventricular, juxtacortical, infratentorial, and spinal lesions with partial gadolinium enhancement, fulfilling dissemination in space and time according to the 2017 McDonald criteria
05/2022 Initial CSF assessment	Lumbar puncture	Mild pleocytosis (8 cells/µL), CSF-restricted oligoclonal bands, eosinophils, erythrocytes and lymphocytes identified on qualitative cytological examination
05/2022 Initial working diagnosis	Diagnostic interpretation	Findings initially considered consistent with MS
05/2022 Initial treatment	First intravenous methylprednisolone (1 g/day for five days)	Partial neurological improvement
06/2022 3 weeks after presentation	New systemic and neurological manifestations	Palpable purpura, inflammatory arthralgia, left foot-elevating paresis (MRC 4/5); the biopsy of the palpable purpura demonstrated dermatitis with neutrophilic infiltration
06/2022 Treatment by suspicion of vasculitis	Second intravenous methylprednisolone (100 mg/day for five days) followed by oral tapering with prednisolone	Resolution of the purpura and arthralgia, left foot-elevating paresis persisted
06/2022 Follow-up evaluation	MRI and neurophysiology	New contrast-enhancing lesion (the corresponding images were not available for inclusion in this report); axonal neuropathy affecting left peroneal and tibial nerves
01/2023 New systemic symptoms	increasing fatigue and severe upper-abdominal pain	Peripheral eosinophilia; gastroscopic biopsy showed interstitial eosinophilic infiltration supporting eosinophilic gastritis in the clinical context; bone marrow eosinophilia (27.4%); an extensive evaluation for secondary, infectious, malignant, and hematological causes of hypereosinophilia did not identify an alternative cause
01/2023 Final diagnosis	Assessment according to 2022 ACR/EULAR classification criteria	Total score 11 points; EGPA diagnosed based on the overall clinical, laboratory, and histopathological findings
01/2023-10/2025 Definitive treatment	intravenous methylprednisolone 40 mg/day for six weeks, followed by oral prednisolone tapering; cyclophosphamide 600 mg intravenous once weekly × 4 weeks, followed by oral cyclophosphamide 100 mg every other day (March–August 2023) and 50 mg every other day (September–October 2023); mepolizumab 300 mg subcutaneously once monthly from March 2023. Maintenance therapy: Mepolizumab 300 mg subcutaneously monthly + prednisolone 2 mg/day	During the subsequent follow-up period, the patient remained clinically stable under maintenance treatment with mepolizumab and low-dose prednisolone, without further clinically apparent neurological or systemic EGPA manifestations.

## Discussion

This case demonstrates how EGPA may initially mimic MS, particularly when CNS lesions and CSF-restricted oligoclonal bands are present. Although demyelinating-appearing lesions and oligoclonal bands are characteristic features of MS, neither finding is disease-specific and both may occur in other inflammatory or autoimmune disorders, including systemic vasculitis. The diagnostic challenge is particularly relevant when neurological manifestations precede the emergence or recognition of the systemic features of EGPA.

EGPA is characterized by eosinophil-rich necrotizing vasculitis and eosinophilic granulomatous inflammation, most commonly involving the respiratory tract, skin, peripheral nervous system, and gastrointestinal tract. CNS involvement is uncommon, but its reported frequency varies substantially among cohorts (6, 7). A Chinese retrospective cohort of 110 patients reported a frequency of 17.3% (7). In that cohort, cerebrovascular manifestations, particularly ischemic lesions, were predominant. Other reported CNS manifestations include hemorrhage, encephalopathy, spinal cord involvement, and, more rarely, inflammatory or demyelinating-like lesions (7, 15–18).

In the present case, the initial neurological presentation was highly suggestive of MS. Brain and spinal MRI demonstrated lesions involving periventricular, juxtacortical, infratentorial, and spinal cord regions, fulfilling dissemination in space. The simultaneous presence of gadolinium-enhancing and non-enhancing lesions provided evidence of dissemination in time, while CSF-restricted oligoclonal bands further supported intrathecal inflammation. These findings were therefore interpreted according to the 2017 McDonald criteria, which were applicable at the time of the patient’s presentation in 2022 (14). However, these criteria were developed primarily for patients with a typical clinically isolated syndrome and require the absence of a better explanation for the clinical presentation. In retrospect, several features already represented important red flags against an isolated diagnosis of MS, including adult-onset asthma, recurrent sinusitis, peripheral eosinophilia, and eosinophils identified on CSF cytological examination.

The subsequent clinical evolution was decisive for diagnostic reassessment. Three weeks after the initial presentation, the patient developed palpable purpura, inflammatory arthralgia, and new peripheral nerve involvement. Peripheral eosinophilia, which had already been present at the initial presentation, persisted. Eosinophilic gastritis and bone marrow eosinophilia were subsequently identified, and the combination of clinical and histopathological findings led to the diagnosis of EGPA. The patient fulfilled the 2022 ACR/EULAR classification criteria for EGPA, providing additional support for the classification of her disease. Importantly, fingolimod had been recommended for the initial working diagnosis of MS but was never initiated. The subsequent disease course therefore occurred under EGPA-directed treatment rather than MS disease-modifying therapy.

CNS involvement in EGPA with demyelinating-appearing lesions has previously been reported. Xu et al. described a patient with EGPA who developed multiple demyelinating-appearing CNS lesions, demonstrating that EGPA may produce radiological findings that overlap substantially with MS (8). The present case adds a different diagnostic perspective. In our patient, the MS-like clinical, radiological, and CSF constellation preceded the development of the characteristic systemic EGPA phenotype. In particular, the presence of CSF-restricted oligoclonal bands further strengthened the initial interpretation as MS. The systemic manifestations became apparent only during subsequent clinical evolution, prompting a fundamental reassessment of the initial diagnosis. Thus, the novelty of this case is not simply that EGPA can produce demyelinating-like CNS lesions, which has been previously described, but that EGPA may initially present with a constellation sufficiently suggestive of MS—including dissemination in space and time and CSF-restricted oligoclonal bands—before systemic vasculitic manifestations become clinically apparent. This temporal evolution illustrates the importance of longitudinal clinical assessment and diagnostic reassessment when apparently demyelinating disease is accompanied by atypical systemic findings.

The diagnostic framework for MS has also evolved since the patient’s initial presentation. The 2024 revision of the McDonald criteria introduced important changes, including the optic nerve as an additional anatomical location and the use of emerging biomarkers such as the central vein sign, paramagnetic rim lesions, and CSF kappa free light chains in selected situations (19). These revised criteria were not available when the patient was evaluated in 2022 and should therefore not be retrospectively applied to the original diagnostic decision. Nevertheless, their emphasis on increasing diagnostic specificity further highlights the importance of excluding alternative inflammatory and systemic disorders when the clinical presentation is atypical.

The present case emphasizes that MRI findings should not be interpreted independently of the systemic phenotype. Adult-onset asthma, chronic or recurrent sinusitis, marked peripheral eosinophilia, purpura, inflammatory arthralgia, peripheral neuropathy, and eosinophilic tissue infiltration should raise suspicion for EGPA even when CNS MRI findings fulfill conventional criteria for MS. In contrast, MS as a primary CNS disorder does not readily explain the combination of eosinophilic systemic inflammation, asthma, peripheral neuropathy, and eosinophilic tissue infiltration.

Neuromyelitis optica spectrum disorders (NMOSD) should also be considered in patients with inflammatory CNS lesions, particularly when the spinal cord is involved (20). In the present patient, serum aquaporin-4 and myelin oligodendrocyte glycoprotein antibodies were negative, and the overall phenotype was not characteristic of NMOSD. The presence of systemic eosinophilic manifestations and peripheral neuropathy instead supported a broader systemic inflammatory process.

A further differential consideration is hypereosinophilic syndrome (HES), particularly given the marked eosinophilia and tissue eosinophilic infiltration. An extensive evaluation for secondary, infectious, malignant, and hematological causes of hypereosinophilia did not identify an alternative cause. Limited T-cell immunophenotyping showed elevated CD4-positive lymphocytes and CD8-positive lymphocytes, with a CD4/CD8 ratio of 2.14. However, molecular testing for FIP1L1::PDGFRA and other myeloid abnormalities, as well as T-cell receptor clonality studies, was not performed. Consequently, myeloid and lymphocytic-variant HES cannot be definitively excluded. Nevertheless, the combination of adult-onset asthma, chronic sinonasal disease, purpura, peripheral neuropathy, marked eosinophilia, and eosinophilic gastrointestinal and tissue involvement favored EGPA as the unifying diagnosis. The patient also fulfilled the 2022 ACR/EULAR classification criteria for EGPA with a score of 11 points. These criteria were considered supportive of the diagnosis but were not used as a substitute for the overall clinical and diagnostic assessment.

The CSF findings represented a particular diagnostic challenge. CSF-restricted oligoclonal bands indicate intrathecal immunoglobulin synthesis but are not specific for MS and may occur in other inflammatory and autoimmune disorders of the CNS (12, 21). In the present case, the IgG index was normal at 0.56, despite the presence of CSF-restricted oligoclonal bands. The coexistence of these findings illustrates that a normal IgG index does not exclude CSF-restricted oligoclonal band formation.

Eosinophils were also identified by cytological examination of the CSF. This represented an unusual and potentially informative finding, although its interpretation requires caution. The eosinophils were identified qualitatively and were not quantitatively documented, and erythrocytes were present in the sample. Peripheral blood contamination could therefore not be completely excluded. Accordingly, CSF eosinophils should be regarded as a supportive but non-specific finding rather than definitive evidence of direct eosinophilic CNS involvement.

The pathophysiological basis of CNS lesions in EGPA remains incompletely understood and may involve a combination of small-vessel vasculitic injury, eosinophil-mediated tissue toxicity, and secondary inflammatory demyelination (12). The presence of both MS-like CNS lesions and systemic eosinophilic inflammation in the present case illustrates the potential overlap between these mechanisms.

ANCA negativity does not exclude EGPA and should not delay consideration of the diagnosis when the clinical phenotype is suggestive. ANCA-positive and ANCA-negative EGPA may differ in their clinical manifestations, with ANCA positivity more commonly associated with certain vasculitic manifestations, while eosinophil-driven organ involvement is frequent in ANCA-negative disease (2, 22). Importantly, neurological involvement may occur in both groups. In the present case, PR3- and MPO-ANCA were negative despite prominent systemic and neurological manifestations. The combination of adult-onset asthma, eosinophilia, chronic sinonasal disease, purpura, peripheral neuropathy, and eosinophilic tissue infiltration provided substantially stronger diagnostic evidence than ANCA status alone.

An additional noteworthy finding was the marked elevation of RF, which increased from 26 IU/mL at initial presentation to 201 IU/mL three weeks later, coinciding with the emergence of palpable purpura, inflammatory arthritis, and new peripheral nerve involvement. Although RF is not a diagnostic marker for EGPA and may be elevated in a variety of inflammatory conditions, recent data suggest that RF positivity may identify a clinically distinct EGPA phenotype. In a recent cohort of 83 patients with EGPA, RF-positive patients more frequently presented with mononeuritis multiplex and experienced systemic relapses more frequently than RF-negative patients; RF positivity was independently associated with a shorter time to systemic relapse after adjustment for MPO-ANCA and treatment intensity (23). The marked increase in RF in our patient occurred in parallel with the development of systemic vasculitic manifestations and peripheral neuropathy, consistent with this recently described phenotype. However, given the single-patient nature of this report, this temporal association should be interpreted cautiously and does not establish RF as a marker of disease activity or causality.

An important aspect of the diagnostic course was that peripheral blood eosinophilia was already present at the initial neurological presentation but was not recognized as diagnostically relevant at that time. The initial work-up was predominantly guided by the neurological presentation and MRI findings, which appeared to fulfill dissemination in space and time and, together with CSF-restricted oligoclonal bands, strongly favored a working diagnosis of MS. Only after eosinophilic infiltration was identified in the gastric biopsy was the earlier laboratory data reviewed retrospectively, revealing that eosinophilia had been present from the beginning. This illustrates the risk of diagnostic anchoring when a plausible neurological diagnosis is established early in the course of a multisystem inflammatory disorder. Conversely, recent reports have also highlighted the importance of reassessing new or atypical manifestations rather than automatically attributing them to a pre-existing systemic diagnosis (24). In the present case, the emergence of purpura, inflammatory arthritis, eosinophilia, and peripheral neuropathy represented a critical diagnostic turning point. More broadly, systematic review of the complete blood count and differential, particularly the eosinophil count, should form part of the evaluation of atypical neuroimmunological presentations. The emergence of asthma, sinonasal disease, skin lesions, peripheral neuropathy, gastrointestinal manifestations, or other systemic inflammatory features should prompt reconsideration of the initial diagnosis and investigation for systemic eosinophilic disorders.

Corticosteroids remain the cornerstone of EGPA treatment, while immunosuppressive or biologic agents such as cyclophosphamide, rituximab, and mepolizumab are used according to disease severity and organ involvement (2). In the present case, the patient received high-dose corticosteroid treatment followed by cyclophosphamide induction and subsequent mepolizumab maintenance therapy. Mepolizumab targets and neutralizes interleukin-5 (IL-5), thereby reducing eosinophil production and survival. More recently, targeting the IL-5 receptor α (IL-5Rα) with benralizumab has emerged as an alternative approach, resulting in profound depletion of circulating eosinophils. In the phase 3 MANDARA trial, benralizumab was non-inferior to mepolizumab for induction of remission in patients with relapsing or refractory EGPA, supporting both IL-5 and IL-5Rα blockade as effective eosinophil-targeted treatment strategies (13, 14). Thus, although these agents differ mechanistically, both may provide a glucocorticoid-sparing therapeutic approach in eosinophil-driven EGPA (25, 26).

In the present case, no further clinically apparent neurological or systemic EGPA manifestations occurred during follow-up, although residual foot-elevating paresis persisted at MRC grade 4/5. The patient remained clinically stable under mepolizumab and low-dose prednisolone. This clinical response supports the diagnosis of EGPA-associated disease but should not be interpreted as definitive evidence of radiological remission, because comprehensive longitudinal MRI follow-up was not available.

## Limitations

Several limitations should be considered when interpreting this case. First, no CNS biopsy was performed; therefore, direct histopathological evidence of CNS vasculitis is lacking. Moreover, neither of the available tissue biopsies demonstrated definitive vasculitis. The gastric biopsy showed prominent interstitial eosinophilic infiltration, supporting eosinophilic tissue involvement, but no histological evidence of vasculitis. Similarly, the biopsy of the palpable purpura demonstrated a pronounced interstitial and partly perivascular neutrophilic infiltrate but no definite vasculitis. Thus, the available histopathological findings do not provide direct evidence of necrotizing vasculitis and should not be interpreted as histological confirmation of EGPA. Nevertheless, the overall clinical and laboratory constellation, including the multisystem eosinophilic phenotype and fulfillment of the 2022 ACR/EULAR classification criteria, supported EGPA as the most coherent unifying diagnosis. The absence of demonstrable vasculitis in the available tissue samples does not by itself exclude EGPA, particularly given the limited tissue sampling and the potentially focal nature of vasculitic lesions. However, this limitation reduces the degree of histopathological certainty regarding the diagnosis. Accordingly, an eosinophilic disorder such as HES, potentially with concomitant MS, cannot be completely excluded.

Second, long-term radiological follow-up was not available. A brain MRI performed three weeks after the initial presentation demonstrated a new right parietal contrast-enhancing lesion; however, the patient subsequently declined further brain and spinal MRI examinations for financial reasons. Consequently, the long-term evolution of the CNS lesions, including the development of new T2 lesions, persistence or regression of existing lesions, and further gadolinium enhancement, could not be assessed. This limits the ability to definitively distinguish EGPA-associated CNS involvement from concomitant MS. Similarly, the subsequent clinical stability should be interpreted primarily as an observation of the patient’s clinical course rather than as evidence of complete radiological remission, given the absence of comprehensive longitudinal MRI assessment.

Third, concomitant MS cannot be completely excluded. The patient had brain MRI lesions initially considered compatible with MS and CSF-restricted oligoclonal bands. Although the subsequent development of systemic manifestations, marked eosinophilia, peripheral neuropathy, and eosinophilic tissue infiltration favored EGPA as the unifying diagnosis, the presence of oligoclonal bands and demyelinating-like MRI lesions leaves open the possibility of a concomitant inflammatory demyelinating disorder. In the absence of definitive histopathological evidence of vasculitis and comprehensive longitudinal MRI follow-up, this possibility cannot be resolved with certainty.

Finally, CSF eosinophils were identified qualitatively by cytological examination, without a documented absolute or percentage eosinophil count. Erythrocytes were also present in the CSF sample, and peripheral blood contamination could therefore not be completely excluded. Accordingly, the CSF eosinophils should not be considered definitive evidence of direct eosinophilic CNS involvement.

## Patient perspective

The patient reported reassurance after establishment of the final diagnosis and substantial improvement following immunosuppressive therapy. Mild residual weakness of the left foot remained but daily activities were largely restored.

## Conclusion

This case illustrates that EGPA may initially present with a clinical, radiological, and CSF profile strongly suggestive of MS, including dissemination in space and time and CSF-restricted oligoclonal bands. The subsequent recognition of eosinophilia, adult-onset asthma, sinonasal disease, purpura, inflammatory arthritis, peripheral neuropathy, and eosinophilic tissue infiltration strongly supported EGPA as the most likely unifying diagnosis.

The case highlights the importance of integrating neurological findings with the broader systemic phenotype and of reassessing an initial diagnosis when new organ manifestations emerge. In patients with apparent inflammatory demyelinating disease, particularly when eosinophilia, adult-onset asthma, sinonasal disease, skin manifestations, or peripheral neuropathy are present, EGPA and other systemic inflammatory disorders should be considered in the differential diagnosis. Longitudinal clinical reassessment and careful attention to apparently nonspecific systemic abnormalities may help prevent diagnostic anchoring and facilitate earlier recognition and treatment of EGPA.

## Data Availability

The original contributions presented in the study are included in the article/supplementary material. Further inquiries can be directed to the corresponding author.
